# 3-Chloro-4-hydroxy­furan-2(5*H*)-one

**DOI:** 10.1107/S1600536809028724

**Published:** 2009-07-25

**Authors:** Na Zhang, Zhen-Yi Wu, Su-Yuan Xie, Rong-Bin Huang, Lan-Sun Zheng

**Affiliations:** a Department of Chemistry, College of Chemistry and Chemical Engineering, Xiamen University, Xiamen 361005, People’s Republic of China; b State Key Laboratory for Physical Chemistry of Solid Surfaces, Xiamen University, Xiamen 361005, People’s Republic of China

## Abstract

In the title compound, C_4_H_3_ClO_3_, mol­ecules are linked *via* O—H⋯O hydrogen bonds into an infinite chain with graph-set motif *C*(6) along the *c* axis.

## Related literature

4-Hydr­oxy-5*H*-furan-2-one (tetronic acid) forms a subclass of β-hydroxy­butenolides with a generic structure, see: Haynes & Plimmer (1960 ▶). A great number of these compounds and their metabolites are found in many natural products and exhibit a wide array of biological properties, see: Sodeoka *et al.* (2001 ▶). For related structures, see: Ma *et al.* (2004 ▶). For hydrogen-bond motifs, see: Bernstein *et al.* (1995 ▶).
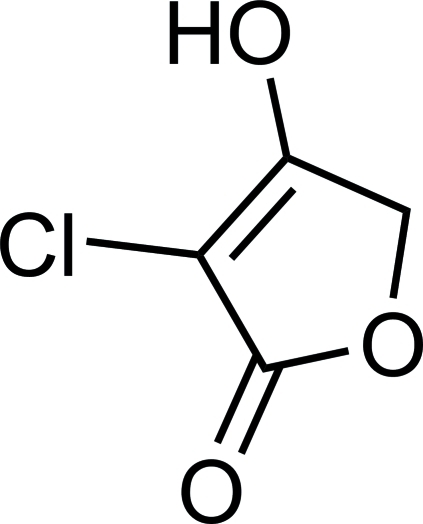


## Experimental

### 

#### Crystal data

C_4_H_3_ClO_3_
*M* *_r_* = 134.51
Orthorhombic, 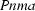

*a* = 12.0437 (6) Å
*b* = 6.5453 (4) Å
*c* = 6.3886 (4) Å
*V* = 503.61 (5) Å^3^
*Z* = 4
Mo *K*α radiation
μ = 0.65 mm^−1^
*T* = 298 K
0.50 × 0.50 × 0.30 mm

#### Data collection

Oxford Gemini S Ultra diffractometer
Absorption correction: multi-scan (*CrysAlis RED*; Oxford Diffraction, 2008 ▶) *T*_min_ = 0.736, *T*_max_ = 0.828
1932 measured reflections
531 independent reflections
500 reflections with *I* > 2σ(*I*)
*R*_int_ = 0.012

#### Refinement

*R*[*F* ^2^ > 2σ(*F* ^2^)] = 0.026
*wR*(*F* ^2^) = 0.067
*S* = 1.17
531 reflections
52 parameters
H atoms treated by a mixture of independent and constrained refinement
Δρ_max_ = 0.25 e Å^−3^
Δρ_min_ = −0.20 e Å^−3^

### 

Data collection: *CrysAlis CCD* (Oxford Diffraction, 2008 ▶); cell refinement: *CrysAlis RED* (Oxford Diffraction, 2008 ▶); data reduction: *CrysAlis RED*; program(s) used to solve structure: *SHELXS97* (Sheldrick, 2008 ▶); program(s) used to refine structure: *SHELXL97* (Sheldrick, 2008 ▶); molecular graphics: *SHELXL97*; software used to prepare material for publication: *SHELXL97* and *publCIF* (Westrip, 2009 ▶).

## Supplementary Material

Crystal structure: contains datablocks I, global. DOI: 10.1107/S1600536809028724/bx2226sup1.cif

Structure factors: contains datablocks I. DOI: 10.1107/S1600536809028724/bx2226Isup2.hkl

Additional supplementary materials:  crystallographic information; 3D view; checkCIF report

## Figures and Tables

**Table 1:** Hydrogen-bond geometry (Å, °)

*D*—H⋯*A*	*D*—H	H⋯*A*	*D*⋯*A*	*D*—H⋯*A*
O2—H2⋯O1^i^	0.80 (3)	1.85 (3)	2.647 (2)	171 (3)

Symmetry code: (i) 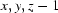.

**Fig. 1.**
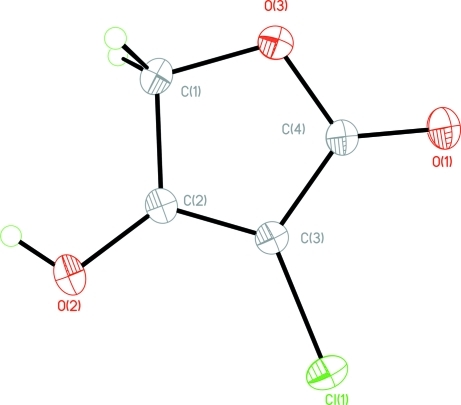
A view of the molecular structure of (I), with the atom-numbering scheme. Displacement ellipsoids are drawn at the 30% probability label and H atoms are shown as small spheres of arbitrary radii.

**Fig. 2.**
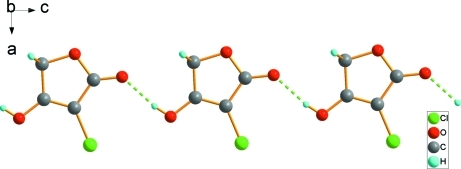
Partial packing view showing the O—H···O interactions (dashed lines) and the formation of a chain parallel to the c axis.

## supplementary crystallographic information

### Comment

4-hydroxy-5*H*-furan-2-one (Tetronic acid) form a subclass of β-hydroxybutenolides with the generic structure (Haynes & Plimmer, 1960). The best known members of this family are vitamin C (ascorbic acid) and pennicillic acid. A great number of these compounds and their metabolites are found in many natural products, which exhibit a wide array of biological properties (Sodeoka *et al.*, 2001). In the present study, the title comound (I) has been determined as product of double-molecular ring closure of monochloroacetic acid which is halo-substituted tetronic acid.

The molecular structure is depicted in Fig. 1. Bond lengths and angles are in good agreement with previous reported for similar compounds (Ma *et al.*, 2004). The crystal structure is stabilized by O—H···O hydrogen bonding and the molecules are linked in an infinite chain along the c axis, with graph-set motifs C(6) through O— H··· O hydrogen bonds (Bernstein *et al., 1995) *(Fig. 2, Table 1).

### Experimental

All reagents and solvents were used as obtained commercially without further purification. To a stirred solution of monochloroacetic acid(2 mmol, 137µ*L*) in 5 mL dry THF is added sodium(1 mmol, 23 mg) under N_2_. After the solution has been stirred at room temperature for 24 h, the resulting pale yellow solution was kept in darkness for four days, yellow well formed block-shaped crystals were obtained.

### Refinement

The aromatic H atoms were generated geometrically (C—H 0.93 Å) and were allowed to ride on their parent atoms in the riding model approximations, with their temperature factors set to 1.2 times those of the parent atoms. The position and *U*_eq_ of the hydroxyl H atom were refined with O—H distance restrained to 0.85 Å.

### Crystal data

**Table:**

C_4_H_3_ClO_3_	*F*(000) = 272
*M**_r_* = 134.51	*D*_x_ = 1.774 Mg m^−^^3^
Orthorhombic, *P**n**m**a*	Mo *K*α radiation, λ = 0.71073 Å
Hall symbol: -P 2ac 2n	Cell parameters from 1627 reflections
*a* = 12.0437 (6) Å	θ = 3.1–28.9°
*b* = 6.5453 (4) Å	µ = 0.65 mm^−^^1^
*c* = 6.3886 (4) Å	*T* = 298 K
*V* = 503.61 (5) Å^3^	Block, yellow
*Z* = 4	0.50 × 0.50 × 0.30 mm

### Data collection

**Table:**

Oxford Gemini S Ultra diffractometer	531 independent reflections
Radiation source: fine-focus sealed tube	500 reflections with *I* > 2σ(*I*)
graphite	*R*_int_ = 0.012
Detector resolution: 16.1903 pixels mm^-1^	θ_max_ = 26.0°, θ_min_ = 3.4°
ω scans	*h* = −14→14
Absorption correction: multi-scan (*CrysAlis RED*; Oxford Diffraction, 2008)	*k* = −7→8
*T*_min_ = 0.736, *T*_max_ = 0.828	*l* = −7→7
1932 measured reflections	

### Refinement

**Table:**

Refinement on *F*^2^	Primary atom site location: structure-invariant direct methods
Least-squares matrix: full	Secondary atom site location: difference Fourier map
*R*[*F*^2^ > 2σ(*F*^2^)] = 0.026	Hydrogen site location: inferred from neighbouring sites
*wR*(*F*^2^) = 0.067	H atoms treated by a mixture of independent and constrained refinement
*S* = 1.17	*w* = 1/[σ^2^(*F*_o_^2^) + (0.0374*P*)^2^ + 0.1215*P*] where *P* = (*F*_o_^2^ + 2*F*_c_^2^)/3
531 reflections	(Δ/σ)_max_ < 0.001
52 parameters	Δρ_max_ = 0.25 e Å^−^^3^
0 restraints	Δρ_min_ = −0.20 e Å^−^^3^

### Special details

**Table:**

Geometry. All e.s.d.'s (except the e.s.d. in the dihedral angle between two l.s. planes) are estimated using the full covariance matrix. The cell e.s.d.'s are taken into account individually in the estimation of e.s.d.'s in distances, angles and torsion angles; correlations between e.s.d.'s in cell parameters are only used when they are defined by crystal symmetry. An approximate (isotropic) treatment of cell e.s.d.'s is used for estimating e.s.d.'s involving l.s. planes.
Refinement. Refinement of *F*^2^ against ALL reflections. The weighted *R*-factor *wR* and goodness of fit *S* are based on *F*^2^, conventional *R*-factors *R* are based on *F*, with *F* set to zero for negative *F*^2^. The threshold expression of *F*^2^ > σ(*F*^2^) is used only for calculating *R*-factors(gt) *etc*. and is not relevant to the choice of reflections for refinement. *R*-factors based on *F*^2^ are statistically about twice as large as those based on *F*, and *R*- factors based on ALL data will be even larger.

### Fractional atomic coordinates and isotropic or equivalent isotropic displacement parameters (Å^2^)

**Table:**

	*x*	*y*	*z*	*U*_iso_*/*U*_eq_	Occ. (<1)
Cl1	0.34253 (4)	0.7500	0.60829 (9)	0.0400 (2)	
O1	0.10530 (15)	0.7500	0.8246 (3)	0.0531 (5)	
O2	0.24683 (15)	0.7500	0.1417 (2)	0.0409 (4)	
O3	0.02339 (12)	0.7500	0.5138 (2)	0.0410 (4)	
C1	0.05689 (17)	0.7500	0.2974 (3)	0.0348 (5)	
H1A	0.0295	0.8706	0.2259	0.042*	0.50
H1B	0.0295	0.6294	0.2259	0.042*	0.50
C2	0.18096 (17)	0.7500	0.3064 (3)	0.0288 (4)	
C3	0.21185 (17)	0.7500	0.5069 (3)	0.0289 (4)	
C4	0.11471 (18)	0.7500	0.6361 (3)	0.0335 (5)	
H2	0.210 (3)	0.7500	0.037 (5)	0.059 (9)*	

### Atomic displacement parameters (Å^2^)

**Table:**

	*U*^11^	*U*^22^	*U*^33^	*U*^12^	*U*^13^	*U*^23^
Cl1	0.0274 (3)	0.0523 (4)	0.0403 (3)	0.000	−0.0084 (2)	0.000
O1	0.0421 (9)	0.0940 (14)	0.0231 (8)	0.000	0.0033 (6)	0.000
O2	0.0376 (8)	0.0614 (11)	0.0238 (8)	0.000	0.0062 (7)	0.000
O3	0.0260 (8)	0.0679 (10)	0.0292 (9)	0.000	0.0011 (6)	0.000
C1	0.0308 (10)	0.0487 (12)	0.0249 (11)	0.000	−0.0042 (8)	0.000
C2	0.0284 (10)	0.0337 (10)	0.0241 (10)	0.000	0.0019 (8)	0.000
C3	0.0251 (10)	0.0355 (10)	0.0261 (11)	0.000	−0.0006 (7)	0.000
C4	0.0299 (10)	0.0460 (12)	0.0247 (11)	0.000	0.0006 (8)	0.000

### Geometric parameters (Å, °)

**Table:**

Cl1—C3	1.702 (2)	C1—C2	1.495 (3)
O1—C4	1.210 (3)	C1—H1A	0.9700
O2—C2	1.318 (2)	C1—H1B	0.9700
O2—H2	0.80 (3)	C2—C3	1.334 (3)
O3—C4	1.349 (3)	C3—C4	1.431 (3)
O3—C1	1.440 (3)		
			
C2—O2—H2	109 (2)	O2—C2—C1	124.82 (18)
C4—O3—C1	109.11 (16)	C3—C2—C1	108.38 (17)
O3—C1—C2	104.08 (16)	C2—C3—C4	109.00 (19)
O3—C1—H1A	110.9	C2—C3—Cl1	128.55 (17)
C2—C1—H1A	110.9	C4—C3—Cl1	122.45 (15)
O3—C1—H1B	110.9	O1—C4—O3	120.0 (2)
C2—C1—H1B	110.9	O1—C4—C3	130.6 (2)
H1A—C1—H1B	109.0	O3—C4—C3	109.43 (17)
O2—C2—C3	126.8 (2)		

### Hydrogen-bond geometry (Å, °)

**Table:**

*D*—H···*A*	*D*—H	H···*A*	*D*···*A*	*D*—H···*A*
O2—H2···O1^i^	0.80 (3)	1.85 (3)	2.647 (2)	171 (3)

Symmetry codes: (i) *x*, *y*, *z*−1.
